# Physical condition, psychological status, and posttraumatic stress disorder among recovered COVID-19 subjects: A mediation analysis

**DOI:** 10.3389/fpsyt.2022.918679

**Published:** 2022-09-06

**Authors:** Kai Zhou, Hongbo Chi, Jing Wang, Yufen Zheng, Juan Pan, Die Yu, Jiaqin Xu, Hongguo Zhu, Jun Li, Shiyong Chen, Xinzhuan Zhao, Xiaomai Wu, Bo Shen, Tao-Hsin Tung, Chengwen Luo

**Affiliations:** ^1^Department of Clinical Laboratory, Taizhou Hospital of Zhejiang Province Affiliated to Wenzhou Medical University, Linhai, China; ^2^Department of Clinical Laboratory, Taizhou Hospital of Zhejiang Province, Zhejiang University, Linhai, China; ^3^Department of Respiratory Medicine, Taizhou Hospital of Zhejiang Province Affiliated to Wenzhou Medical University, Linhai, China; ^4^Evidence-Based Medicine Center, Taizhou Hospital of Zhejiang Province Affiliated to Wenzhou Medical University, Linhai, China

**Keywords:** physical condition, psychological status, PTSD, COVID-19, mediation analysis

## Abstract

The physical condition of individuals who contracted COVID-19 had a profound influence on mitigating the physical and psychological impact of the disease and the symptoms of posttraumatic stress disorder (PTSD). Little attention has been focused on the influence of physical condition on PTSD among recovered COVID-19 subjects. This study explored the relationship between physical and psychological status and PTSD and the potential mechanisms. Questionnaires were completed by 73 (50.7%, 73/144) COVID-19 recovered subjects who were diagnosed in Taizhou, Zhejiang, China. We conducted a face-to-face survey from January 17 to March 10, 2020. The mediation analysis approach was applied in this research. Our data show that recovered COVID-19 subjects who were in better physical condition exhibited fewer psychological problems [B (95%CI), (−1.65 −3.04, −0.26)] and lower PTSD [B (95%CI), −6.13 (−9.43, −2.83)]. In addition, the worse the psychological status of recovered COVID-19 subjects was, the stronger the PTSD (B [95%CI], 0.58 [0.02, 1.14]). Moreover, psychological status could significantly mediate the impact of physical condition on PTSD (_β_1_θ2_ = −0.87). Together, COVID-19 recovered subjects who have better physical condition could decrease their PTSD, and the worse the physical condition of COVID-19 recovered subjects would increase their psychological problems. Our finding about psychological status could significantly mediate the impact of the physical condition on PTSD might be useful for medical institutions and the government seeking to help with the follow-up rehabilitation training of recovered COVID-19 subjects.

## Introduction

The spread of SARS-CoV-2, regarded as a public health emergency of international concern, continues. In the past 2 years, the number of confirmed COVID-19 cases has exceeded 570 million worldwide, and the number of deaths has exceeded 6.3 million (https://covid19.who.int/).

The sequelae of recovery from acute COVID-19 have been widely reported (1–3). The psychological status and posttraumatic stress disorder (PTSD) in recovered COVID-19 subjects have also received widespread attention (4–6). According to a new systematic study, the majority of patients recovered good physical functioning, but more patients reported anxiety or depression at 12 months of follow-up than at 6 months (1). In addition, the recovered COVID-19 subjects at the 1-year follow-up also reported that the most common psychiatric disorder was anxiety, followed by depression and PTSD in two small-sample studies (7, 8). People with poor mental health are more likely to suffer from PTSD (9), and anxiety/depression can lead to reduced health-related quality of life (HQoL) (10). Thus, more attention needs to be focused on COVID-19 survivors' mental health, particularly in the early stages of PTSD, when the care could play an important role in the recovery of physical and psychological health.

Anxiety disorders are characterized by excessive fear (and avoidance), usually in response to a specific object or situation. The ventromedial pre-frontal cortex (vmPFC) is involved in a variety of social, cognitive, and emotional functions that are usually disrupted in psychiatric disorders (11). More than half of patients with PTSD have psychiatric co-morbidities, such as mood or anxiety (12).

Post-traumatic stress disorder (PTSD) is a disorder that is clearly defined as a maladaptive response to a traumatic event (13). The current neurobiological understanding of PTSD highlights abnormalities in fear learning, threat detection, executive function and emotion regulation, and contextual processing during the development of PTSD (14). It has been suggested that PTSD is underpinned by deficits in fear extinction memory (15–17). In patients with PTSD, the size and activity of the ventral medial pre-frontal cortex (vmPFC) are related to the degree of fear extinction deficit (18), as demonstrated by a negative correlation between PTSD symptom severity and vmPFC activation (19, 20). Other studies have shown that PTSD patients usually show increased amygdala activity and decreased medial pre-frontal cortex (mPFC) activity in symptom provocation studies (21–25). The amygdala is a key limbic structure involved in emotional responses, and regions of the vmPFC exert inhibitory effects, and effective regulation of amygdala responses is achieved by the pre-frontal cortex (PFC), especially the ventral medial PFC (vmPFC) (26, 27). The studies of relationship between psychological status and PTSD-related neurocircuitry have not been reported in COVD-19.

In this study, the European Quality of Life (EuroQol)-5 Dimensions-5 level (EQ-5D-5L), 12-item General Health Questionnaire (GHQ-12) and Event Impact Scale (IES-R) were used to comprehensively assess the physical status, psychological status and PTSD of recovered COVID-19 subjects 1 year after hospital discharge. Hypothesizing that physical status can directly affect PTSD and indirectly affect PTSD through mental health, we established a structural equation to provide better personalized treatment options for recovered COVID-19 subjects.

## Materials and methods

### Study design and population

A predictive questionnaire-based survey was used in this study to investigate the relationships between recovered COVID-19 subjects physical, psychological status and PTSD, and the mediating role of the psychological status in these relationships. We followed up 144 subjects for 1 year after they were discharged from Taizhou Hospital where they were formerly confirmed with COVID-19. The following subjects were excluded: (1) 42 subjects who refused to participate or were lost to follow-up, (2) 6 subjects who could not be contacted, and (3) 23 subjects who lived outside Taizhou. We used multiple linear regression model to calculate the effects of predictors on the outcome, assuming the effect size level of 0.25, the significance level of 0.05, the power of 0.80, and the number of potential predictors of 6 (physical condition, psychological status, age, sex, BMI, and symptom). The target sample size was 62 participants. We allowed for a 10% participant dropout (reluctance to participate in surveys) and selected 69 participants as a conservative sample size. The sample size was calculated using the software G.Power 3.1.9.6.

### Questionnaires

All eligible recovered COVID-19 subjects met with researchers at Taizhou Enze Hospital of Enze Medical Center on February 9 or February 10, 2021. If they missed their appointments, subjects had the opportunity to reschedule the appointment for February 13, 2021. All participants were interviewed in person by trained doctors and asked to complete a series of questionnaires, including the EQ-5D-5L, GHQ-12, and PTSD. New symptoms within 1 year, such as fatigue or muscle weakness, dyspnoea, and joint pain, were recorded.

The EQ-5D-5L is a validated quality-of-life (QoL) questionnaire that evaluates subjects' QoL through five factors: mobility, self-care, daily activities, pain or discomfort, anxiety or depression (28). The classification of each factor was divided into five levels, ranging from 1 to 5, and we obtained results similar to 11111, 11112. Then, according to the Asian EQ-5D-5L score calculator (29), the EQ-5D-5L scores were obtained, with 1 being the highest value. To measure the mental health of recovered COVID-19 subjects, we used the GHQ-12 (30), which included 12 items that assessed symptoms related to psychological distress and general functioning (31). The binary scoring method was used [0-0-1-1], the first two answers and last two answers were counted as 0 and 1, respectively, and the total score range was 0–12, with 3 as the cut-off value (α = 0.82) (32, 33). PTSD was assessed by using the Event Impact Scale (IES-R) (34). Our questionnaire included five items, which were assessed on a scale of 0–4 (from “not at all” to “extremely”) with a total score of 0–20 (α = 0.77). All participant questionnaires were assessed by trained professionals referencing the above criteria.

### Mediation analysis

Mediation occurs as part of a hypothetical causal chain of events: the exposure affects the mediator, which then influences the outcome variable. Mediation analysis has been widely used to understand the mechanism of exposure on an outcome, which is an attractive method for studying disease causal pathways. Researchers can obtain direct and indirect effects *via* an intermediate variable by partitioning the total effect of an exposure on an outcome (35, 36). Mediation regression models are often used to assess mechanism by which the exposure has an effect on the outcome. In this research, we focused on a continuous exposure (*X*), a continuous mediator (*M*), and a continuous outcome (*Y*). We adopted the following simple models for mediation analysis:


(1) $$\begin{array}{r} Y=\psi_{0}+\psi_{1}X+b_{1}^{T}Z, \end{array}$$

(2) $$\begin{array}{r} M=\beta_{0}+\beta_{1}X+b_{2}^{T}Z, \end{array}$$

(3) $$\begin{array}{r} Y=\theta_{0}+\theta_{1}X+\theta_{2}M+b_{3}^{T}Z, \end{array}$$

where Equation (1) describes the relationship between *X* and *Y*; Equation (2) characterizes the relationship between *X* and *M*; Equation (3) summarizes the relationship between *X*, *M*, and *Y*; ψ_1_ is the total effect of *X* on *Y*; β_1_ is the effect of *X* on *M*; θ_1_ is the direct effect of *X* on *Y*; θ_2_ is the effect of *M* on *Y*; *Z* denotes the potential confounders; *b*_1_, *b*_2_, and *b*_3_ are the effects of *Z* on *X*, *M*, and *Y*, respectively; and ψ_0_, β_0_, and θ_0_ are the intercept terms.

Mediation is typically tested using a four-step procedure based on the regression-based modeling approach (37–39). Step 1 identifies the relationship between the exposure *X* and the outcome *Y*. Step 2 is to identify the relation of exposure *X* and mediator *M*. The third and fourth steps are to regress the outcome *Y* on both *X* and *M*. Here, we adopted the joint test for intermediate effects (40).

### Statistical analysis

**Figure 1:**
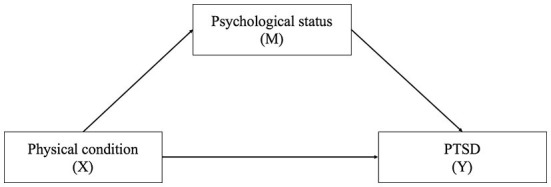
The directed acyclic graph describes the relation among physical condition, psychological status, and PTSD. X, exposure; M, mediator; Y, outcome.

Our primary interest was to study the relationship between physical condition (*X*) and posttraumatic stress disorder (PTSD, *Y*) and whether psychological status (*M*) could mediate the relationship. The directed acyclic graph describes the above relations is shown in Figure 1. We applied a regression model in the mediation analysis. All analyses were carried out using R software, version 4.1.0 (R Project for Statistical Computing).

First, frequencies and percentages were presented for the baseline characteristics of recovered COVID-19 subjects. Second, we conducted a correlation matrix that included physical condition, psychological status, and PTSD. Third, the three regression models (i.e., Equations 1–3) were adopted to carry out mediation analyses (41, 42). The first model estimated the effects of exposure on the outcome. In the second model, the moderating effect of exposure on the outcome was estimated. In the third model, the moderating effect of exposure on the outcome after controlling the mediator and the effect of the mediator on the outcome after controlling exposure were estimated. In addition, the four-step procedure was applied to test for the mediation effect of the mediator between the exposure and the outcome, including the significance of the following relationships: (1) exposure and outcome (*X*→*Y*), (2) exposure and mediator (*X*→*M*), (3) mediator and outcome while controlling for exposure (*M*→*Y*|*X*); and (4) exposure and outcome while controlling for the mediator (*X*→*Y*|*M*). All of the above relationships were adjusted for potential confounders *Z*. In this process, a joint test method was adopted to determine whether variable *M* was a mediator between the exposure and the outcome. Moreover, the coefficients and 95% confidence intervals (CIs) were calculated. A variable with a *P-value* < 0.05 or 95% CI not including zero was considered statistically significant.

## Results

### Characteristics of the study participants

A total of 73 recovered COVID-19 subjects participated in this study. The subjects' basic information, including age, sex, BMI, and symptoms is summarized in Table 1. The average age of the individuals was 47.3 ± 13.8 years, and most were under 45 years, accounting for 46.6%. There were 39 males (53.4%) and 34 females (46.6%). More than half of the individuals had BMI levels below 25 kg/*m*^2^ (58.9%). In addition, the proportion of recovered subjects with mild symptoms of COVID-19 was 74, and 26% had severe symptoms. Moreover, we found that 38.4, 24.7, and 26% of participants had symptoms of tiredness, breathing with difficulty, and arthralgia, respectively.

**Table 1:** Baseline characteristics of COVID-19 recovered subjects (*n* = 73).

**Characteristics**	**Category**	**Sample**	
		**Number**	**Percentage (%)**
Age (years)	<45	34	46.6
	45–60	28	38.3
	≥60	11	15.1
Sex	Male	39	53.4
	Female	34	46.6
BMI (kg/*m*^2^)	<25	43	58.9
	≥25	30	41.1
Symptom	Mild	54	74.0
	Severe	19	26.0
Tiredness	0 (No)	45	61.6
	1 (Yes)	28	38.4
Breath with difficulty	0 (No)	55	75.3
	1 (Yes)	18	24.7
Arthralgia	0 (No)	54	74.0
	1 (Yes)	19	26.0

### Correlations between the main study variables

The results of Pearson correlation are given in Table 2. Physical condition was negatively correlated with PTSD (r = −0.40, *P-value* <0.001) and psychological problems (r = −0.25, *P-value* <0.05). Psychological status was significantly correlated with PTSD (r = 0.34, *P-value* <0.01). Overall, the results of the correlation analysis show that the pairwise combinations of the three variables are significant and that there was a correlation between physical condition and psychological status, and PTSD.

**Table 2:** Descriptive statistics and Pearson correlations among physical condition, psychological status, and PTSD (*n* = 73).

**Variables**	**M**	**SD**	**1. Physical condition**	**2. Psychological status**	**3. PTSD**
1. Physical condition	0.88	0.12	1.00		
2. Psychological status	0.32	0.70	−0.25*	1.00	
3. PTSD	1.15	1.78	−0.40***	0.34**	1.00

^*^p < 0.05; ^**^p < 0.01; ^***^p < 0.001.

M, mean; SD, standard deviation; PTSD, post-traumatic stress disorder.

### Testing for the mediation model

The results of the mediation analyses for the relationships among physical condition, psychological status, and PTSD, adjusting for age, sex, and occupation, are presented in Table 3. We first conduct mediation regression analysis based on the three models Equation (1)–(3). Based on the regression results, we carry out the four-step procedure to test for the mediation effect of psychological status between physical condition and PTSD. In step one, the effect of physical condition on PTSD was significant (*P–value* <0.001). Participants with a better physical condition had less PTSD (B = −6.13, 95%CI: −9.43 to −2.83). Henc e, the physical condition was a significant factor influencing PTSD. In step two, we found that individuals who had better physical conditions had fewer psychological problems (B = −1.65, 95%CI: −3.04 to −0.26). The effect of physical condition on psychological status was significant (*P-value* <0.05).

**Table 3:** Mediation regression analyses for the relationships between physical condition, psychological status, and PTSD.

**Variable**	**Model 1**		**Model 2**		**Model 3**	
	**B**	**95%CI**	**B**	**95%CI**	**B**	**95%CI**
**Independent variable**						
Physical condition	−6.13***	−9.43 to −2.83	−1.65*	−3.04 to −0.26	−5.17**	−8.52 to −1.81
**Mediator**						
Psychological status					0.58*	0.02 to 1.14
**Controlled variable**						
Age (45 to 60)	−0.09	−0.96 to 0.78	−0.11	−0.48 to 0.25	−0.02	−0.87 to 0.83
Age (≥60)	0.03	−1.27 to 1.34	−0.12	−0.66 to 0.43	0.10	−1.16 to 1.37
Sex (Female)	−0.67	−1.51 to 0.17	−0.24	−0.59 to 0.11	−0.52	−1.35 to 0.30
BMI (≥25)	−0.53	−1.41 to 0.34	−0.03	−0.40 to 0.34	−0.51	−1.37 to 0.34
Symptom (Severe)	0.15	−0.90 to 1.20	0.15	−0.29 to 0.59	0.06	−0.96 to 1.09

^***^P–value < 0.001; ^**^P–value < 0.01; ^*^P–value < 0.05. The outcome of Model 1 and 3 was PTSD; the outcome of Model 2 was psychological status.

B, standardized beta regression coefficient; CI, confidence interval.

In step three, the influence of psychological status on PTSD was also significant after controlling for physical condition, age, sex, BMI, and symptoms (B = 0.58, 95%CI: 0.02–1.14, *P-value* <0.05). In step four, the effect of physical condition on PTSD decreased but remained significant (B = −5.17, 95%CI: −8.52 to −1.81, *P-value* <0.01) after controlling for psychological status, age, sex, BMI, and symptoms. The joint test indicated that the mediation effect of psychological status on the relationship between physical condition and PTSD was significant (*P* = max(*P*_β_1__, *P*_θ_2__) < 0.05). This implies that psychological status could significantly mediate the effect of physical condition on PTSD.

## Discussion

The research aimed to explore the relationship between physiological, psychological states, and PTSD and its underlying mechanism. We focused on individuals who had recovered from COVID-19, and we surveyed them regarding the above aspects 1 year after hospital discharge. We found that individuals' physical condition had an impact on their physical and psychological status, and this mediated symptoms of PTSD. Therefore, how physical condition impacts PTSD and the QoL of recovered COVID-19 subjects should interest governments and hospitals. In this research, we found that recovered COVID-19 subjects who were in better shape physically had better psychological status and lower PTSD. In addition, the worse the psychological status of recovered COVID-19 subjects was, the stronger the PTSD symptoms. Moreover, psychological status could significantly mediate the impact of physical condition on PTSD. To the best of our knowledge, this paper is one of the few studies on the influence of physical condition on PTSD among recovered COVID-19 subjects.

Viral respiratory illness is associated with acute and long-term psychopathological outcomes in recovered COVID-19 subjects (43), according to previous studies of severe acute respiratory syndrome (SARS). During follow-up of 1 to 50 months, SARS survivors reported psychiatric symptoms, including PTSD, depression, panic attacks, and obsessive-compulsive disorder (OCD) (44–46). In a 1-year follow-up study of PTSD and its related factor analysis in recovered Chinese COVID-19 subjects, approximately 9.28% reported PTSD symptoms, and those in poor health patients had higher PTSD scores (β = 0.184) (47), but the study did not adequately assess or quantify the health status of subjects. The EQ-5D-5L index can distinguish adolescents with different degrees of PTSD (48). By quantifying QoL in five dimensions through the EQ-5D-5L, our study also found that health status with higher EQ-5D-5L scores was associated with lower PTSD, indicating that physical condition was a significant influencing factor for PTSD. However, the mechanisms of PTSD caused by physical condition are still unclear in recovered subjects with long-term COVID-19 syndrome.

Traumatic events studies have found that higher GHQ-12 scores (poorer mental health) correlated with higher DTS-8 scores (greater likelihood of developing PTSD) (31, 49, 50); in the HIV patients being stigmatized, the incidence of PTSD was 27.4%, and independent predictors included the presence of general psychopathology (51). This implies that psychological status could significantly mediate the effect of physical condition on PTSD (_β_1_θ2_ = −0.87). The high prevalence of anxiety and depressive symptoms among COVID-19 survivors at 6 months may be predictive of mood or anxiety disorders and posttraumatic stress disorder. Targeted psychological interventions can help curb psychological sequelae and promote post-traumatic growth (52). Therefore, it is necessary to give psychological intervention and study the potential mechanism of PTSD patients after COVID-19 recovery.

Aberrant fear conditioning is widely recognized as an underlying mechanism in various psychiatric disorders, particularly post-traumatic stress disorder (53). Fear conditioning is a phenomenon mainly involved in the central nervous system, especially the pre-frontal cortical-amygdala-hippocampal circuit (54, 55). Under normal conditions, the pre-frontal cortex recognizes security cues from the environment and exerts inhibitory control over sympathetic excitatory subcortical circuits *via* vagal control. In contrast, in situations of threat and uncertainty, pre-frontal inhibitory regulation attenuates vagal control (56). In the NVI-f model, cascading higher cognitive structures, particularly the pre-frontal cortex, trigger neurological visceral fear responses by modulating sympathetic and parasympathetic projections of heart-related dynamics (57). Vagally mediated heart rate variability (HRV), an indicator of parasympathetic activity, is thought to possibly reflect their ability to fear to acquire and abate learning (58). Consistent with this, low levels of HRV are associated with a higher prevalence of anxiety disorders and deficits in safety learning (59). Pre-frontal vagal control activation was reduced in patients with significantly increased heart rate, whereas vagal control activation was increased in patients with slower heart rate acceleration. The above suggests an important structural role of frontal circuits in fear extinction and emotional learning ability. Functional neuroimaging evidence suggests that activity in the vmPFC is involved in the positive affective processing of safety signals, including flexible control of fear and other emotions (60, 61). It was further found that in PTSD patients, the size and activity of the vmPFC correlated with the degree of fear extinction deficits. Taken together, this suggests that an in-depth study of the pre-frontal cortex, and especially vmPFC, function in COVID-19 rehabilitated PTSD patients is warranted.

Therefore, this study informs clinicians that while attending to individuals' physical health, they also need to be concerned about their mental health. Our findings indicate the benefits of psychological intervention in treating the challenges to mental health brought about by the pandemic and how to conduct a psychological intervention. We also found that social isolation is associated with intense psychological distress (62), but the psychological flexibility of parents is a mediator of PTSD in children (31). Of course, in addition to medical care and family support, society as a whole needs to give attention and care to individuals who may experience long-term PTSD following recovery from COVID-19. We recommend assessing the psychopathology of recovered COVID-19 subjects and monitoring changes over time to reduce the social burden of the disease.

## Study limitations

Several limitations should be considered when interpreting the results of this study. First, the study population that we selected was the total recovered COVID-19 subjects in one city. However, there might be differences between cities and regions. In addition, the sample size of our analysis was not large enough, and the statistical power might be affected. Hence, to further identify the underlying mechanism, the generalization and external validity should be further studied. Second, the Hawthorne effect was inevitable due to conducting the survey face to face. Third, considering that we did not control for all of the variables that might be associated with PTSD in this study, the relationship between PTSD and physical condition and psychological status could have been affected by underlying diseases. Fourth, we conducted this study based on a single-time measurement, which might not reflect long-term exposure to physical conditions, psychological status, and PTSD. It is of great importance for future studies to consider larger sample sizes and longitudinal studies across a wider range of regions and populations.

## Conclusion

The psychiatric consequences of SARS-CoV-2 infection could be caused either by an immune response to the virus itself or by psychological stressors such as social isolation (63), the psychological effects of a new and potentially fatal disease, worries of infecting others, and stigmatization. In summary, our study showed that the worse the physical condition of recovered COVID-19 subjects was, the greater their psychological problems, which in turn raised their levels of PTSD. This may be due to structural and functional changes in the brain that cause vmPFC dysfunction, resulting in PTSD symptoms after recovery. Overall, these findings about psychological status could significantly mediate the impact of the physical condition on PTSD might be useful for medical institutions and the government seeking to assist recovered COVID-19 subjects with their rehabilitation, and may contribute to provide theoretical basis for further neurocircuitry studies and drug targets in patients with COVID-19 PTSD.

## Data availability statement

The raw data supporting the conclusions of this article will be made available by the authors, without undue reservation.

## Ethics statement

The study was approved by the Ethics Committee of Taizhou Hospital of Zhejiang Province. Informed consent was obtained from each subject. The minors' participation in the study was approved by their parents and/or legal guardians.

## Author contributions

T-HT and BS supervised the project. T-HT and CL designed the study. KZ and CL organized the data, established mediation analysis model, and wrote the manuscript. KZ, HC, JW, YZ, JP, DY, JX, HZ, JL, SC, and XZ collected clinical data and provided clinical supervision. XW assisted in completing the face-to-face follow-up. All authors contributed to the article and approved the submitted version.

## Funding

This work was supported by Zhejiang Provincial Medical and Health Science and Technology Program (2019RC089).

## Conflict of interest

The authors declare that the research was conducted in the absence of any commercial or financial relationships that could be construed as a potential conflict of interest.

## Publisher's note

All claims expressed in this article are solely those of the authors and do not necessarily represent those of their affiliated organizations, or those of the publisher, the editors and the reviewers. Any product that may be evaluated in this article, or claim that may be made by its manufacturer, is not guaranteed or endorsed by the publisher.
